# Assessing Adherence and Identifying Barriers to Colorectal Cancer Screening in the Adult General Populations of Saudi Arabia: A Nationwide Cross-Sectional Study

**DOI:** 10.3390/ijerph22091386

**Published:** 2025-09-05

**Authors:** Ibrahim A. Alamer, Rayan A. Altuwaijri, Salman F. Alfraih, Bader Shabib Alotaibi, Mohanad A. Alqahtani, Sultan Alnashmi Alqasim, Khalid A. Bin Abdulrahman

**Affiliations:** 1 College of Medicine, Imam Mohammad Ibn Saud Islamic University (IMSIU), Riyadh 13317, Saudi Arabia; 442022047@sm.imamu.edu.sa (I.A.A.); 442020004@sm.imamu.edu.sa (R.A.A.); 442019158@sm.imamu.edu.sa (S.F.A.); 442019850@sm.imamu.edu.sa (B.S.A.); 442016025@sm.imamu.edu.sa (M.A.A.); 2 College of Medicine, King Saud bin Abdulaziz University for Health Sciences (KSAU-HS), Riyadh 11481, Saudi Arabia; alqassim372@ksau-hs.edu.sa; 3 Department of Medical Education, College of Medicine, Imam Mohammad Ibn Saud Islamic University (IMSIU), Othman Bin Affan Rd., P.O. Box 7544, Riyadh 13317, Saudi Arabia

**Keywords:** colorectal cancer screening, Saudi Arabia, screening barriers, public health, fecal occult blood test, colonoscopy utilization, health awareness

## Abstract

Background: Colorectal cancer (CRC) is a leading cause of cancer-related morbidity and mortality worldwide. Despite its preventability through early screening, uptake remains suboptimal in many countries, including Saudi Arabia. This study aimed to assess adherence to CRC screening guidelines and identify barriers among the adult population in Saudi Arabia. Methods: A nationwide cross-sectional study was conducted using a self-administered online questionnaire targeting individuals aged ≥ 40 years across all Saudi regions. Data on demographic characteristics, knowledge, attitudes, screening practices, and perceived barriers were analyzed using SPSS v26, with significance set at *p* < 0.05. Results: Of the 573 eligible participants, only 12.7% had undergone colonoscopy and 19.5% reported having completed a fecal occult blood test (FOBT). The most frequently cited barriers were the absence of symptoms (49.9%), fear of the procedure (36.6%), and lack of knowledge (35.3%). Notably, 84.5% indicated they would undergo screening if recommended by a physician. Regional disparities were evident, with participants from the Southern region significantly more likely to undergo FOBT (OR = 8.97, *p* < 0.001). Awareness was generally low, with over half of the participants rating their CRC screening knowledge as 1 out of 10. Conclusions: This study revealed a concerningly low rate of colorectal cancer screening among adults in Saudi Arabia. Efforts to increase screening rates should prioritize raising awareness, correcting misconceptions, and encouraging non-invasive screening methods. Establishing a nationwide screening initiative could help close existing gaps and support earlier detection of colorectal cancer.

## 1. Introduction

Cancer, the second leading cause of both death and incidence worldwide, represents a major global health issue in terms of morbidity and mortality. According to the International Agency for Research on Cancer (IARC), in 2022, there were approximately 20 million new cancer diagnoses and 9.7 million cancer-related deaths [1]. Colorectal cancer (CRC) is the third most commonly diagnosed cancer worldwide, after lung and breast cancer, and ranks second in terms of cancer-related mortality worldwide [2,3]. According to the IARC’s 2022 GLOBOCAN estimates, the worldwide incidence of colorectal cancer was approximately 1.9 million new cases and 0.9 million deaths [1]. However, around 90% of CRCs are adenocarcinomas, and almost half of the patients with CRC will develop metastatic CRC (mCRC) [4].

In 2022, the IARC reported 28,113 new cancer cases and 13,399 cancer-related deaths in Saudi Arabia. However, when it comes to CRC in Saudi Arabia, it was estimated that there are approximately 3750 new cases and 1883 deaths, making it the most common cancer in males and the third most common in females. The age-standardized incidence rate (ASIR) is 14.5 per 100,000 population and the age-standardized mortality rate (ASMR) is 7.3 per 100,000 population in the same year. Thus, patients with CRC have around a 50% mortality rate [5].

CRC risk factors can be classified as modifiable and non-modifiable categories. Modifiable risk factors contribute to 60–65% of CRC cases and can be influenced by individual behaviors and environmental factors. These include obesity, physical inactivity, high consumption of red and processed meats, low intake of dietary fiber, insufficient consumption of dairy products, smoking, and alcohol consumption [6]. In contrast, non-modifiable risk factors, which account for approximately 25–30% of CRC cases, include age (with risk increasing after the age of 50), personal or family history of CRC or colorectal polyps, inflammatory bowel diseases (such as ulcerative colitis and Crohn’s disease), and hereditary conditions like familial adenomatous polyposis and Lynch syndrome. These factors cannot be altered through lifestyle changes [6,7].

In Saudi Arabia, the Ministry of Health recommends annual immunochemical stool testing for individuals aged 45–75 years at average risk, with colonoscopy reserved for those at higher risk or with positive stool tests. Despite the availability of these recommendations, population-level uptake has remained low. Previous regional studies have primarily focused on small subgroups or lacked a nationwide perspective [8,9], leaving a knowledge gap regarding adherence and barriers to CRC screening across all Saudi regions.

Colorectal cancer (CRC) screening aims to detect early-stage CRC and to identify and excise adenomas and Sessile Serrated Lesions (SSLs) by various screening modalities, including colonoscopy, sigmoidoscopy, CT colonography, and fecal immunochemical tests. These modalities effectively reduce both the incidence and mortality rates in early-stage CRC [3,4,5,6,7,8,9,10].

This study aims to assess the adherence to CRC screening guidelines among high-risk populations in Saudi Arabia and identify the key barriers that hinder effective participation in these screening programs. For this study, ‘high-risk’ was operationally defined as adults aged 40 years and above who were eligible for CRC screening, were willing to participate, and had no prior diagnosis of colorectal cancer. By investigating factors such as awareness, cultural attitudes, accessibility, and socioeconomic status, this study seeks to offer essential details that may help inform public health policies and enhance the adoption of colorectal cancer screening, eventually allowing early identification and better management of this disease in high-risk populations.

## 2. Materials and Methods

### 2.1. Study Design and Participants

A cross-sectional analytic study was conducted across all regions of Saudi Arabia from 3 May 2025 to 30 June 2025. All Saudi regions have comparable access to non-invasive CRC screening (FOBT/FIT) through public primary healthcare centers (PHCs), where tests are available free of charge under the national CRC screening program. The study targeted the general population aged above 40 years, irrespective of nationality, to evaluate adherence to and barriers against colorectal cancer (CRC) screening. Participants with a previous diagnosis of CRC and those unwilling to participate were excluded.

### 2.2. Ethical Considerations and Sample Size

The study protocol was approved by the Institutional Review Board (IRB) of Imam Mohammad Ibn Saud Islamic University under the reference number HAPO-01-R-061 (project number 793–2025; approval date, 30 April 2025). Participation was voluntary, and electronic informed consent was obtained from each participant before inclusion. All participant data were treated confidentially and securely, with access restricted to the research team. The required sample size was calculated using the Raosoft sample size calculator to be at least 385 participants, based on a 95% confidence level and a 5% margin of error, to ensure adequate power for statistical analysis.

### 2.3. Study Tool and Data Collection

Data were collected using a self-administered electronic questionnaire via Google Forms, distributed randomly across all regions of Saudi Arabia. Participants were primarily recruited through institutional mailing lists (48.5%), social media platforms such as Twitter and WhatsApp (37.4%), and direct peer-to-peer snowball sharing (14.1%). Although online surveys may overrepresent digitally literate and health-conscious individuals, the use of diverse recruitment strategies helped mitigate this bias by reaching participants across different demographic groups. Duplicate responses were controlled through Google Form settings.

A validated survey tool developed by Allam et al. [11] was used to assess participants’ knowledge, attitudes, and behaviors regarding CRC screening. The questionnaire included sections on whether the respondent had undergone a colonoscopy for CRC screening, as well as perceived barriers to screening. It also gathered demographic data, including age, gender, region of residence, monthly income, and education level. The questionnaire had previously demonstrated robust psychometric properties with Cronbach’s alpha values >0.80. Given that our study did not introduce modifications to the items, and that the primary focus was on population-level screening behaviors rather than instrument development, we retained the validated tool without recalculating Cronbach’s alpha. This approach is consistent with practices in large-scale survey research where the instrument has been validated in comparable populations.

### 2.4. Data Analysis

The statistical analysis was performed using SPSS (IBM version 26). Categorical variables were analyzed and presented as frequencies and percentages. Mann–Whitney and Kruskal–Wallis tests were used to assess the association of CRC screening knowledge with demographic variables, and results were expressed as medians, interquartile ranges, and *p*-values. A *p*-value of < 0.05 was considered statistically significant. Regression analyses were conducted at both univariable and multivariable levels, with results expressed as odds ratios (OR) and 95% confidence intervals (CI). The multivariable logistic regression model was adjusted for age, gender, education, income, and region to account for potential confounding. Model fit was assessed using the Hosmer–Lemeshow goodness-of-fit test, and multicollinearity was checked using variance inflation factors (VIFs). These steps ensured the robustness and validity of the reported associations.

## 3. Results

### 3.1. Demographic Information

The overall response rate was 47.3%, as 573 of 1210 individuals who accessed the survey met the inclusion criteria and completed the questionnaire (age ≥ 40 years and consent to participate). The majority were aged 40–49 years (51.5%), with a slightly higher proportion of males (52.4%) Most respondents were employed and held a university-level education (Table 1).

### 3.2. Medical History

Most participants (87.3%) reported no first- or second-degree relatives with colorectal cancer, and 83.6% had no chronic gastrointestinal diseases. Among those with GI history, intestinal problems were most common (64.4%). Notably, 89.4% had never discussed CRC with a healthcare provider (Table 2).

### 3.3. Knowledge and Attitude Towards Colorectal Cancer (CRC)

A significant proportion of participants (52.0%) rated their CRC screening knowledge as 1 out of 10. The most commonly identified benefit of early screening was early detection (57.4%), and 66.1% believed screening is necessary even without symptoms. However, 81.7% reported not knowing how to screen. Endoscopy was the most recognized method (41.0% of those aware). Support for CRC screening after age 45 was highest among those who rated it 10 out of 10 (51.0%) (Table 3).

**Figure 1:**
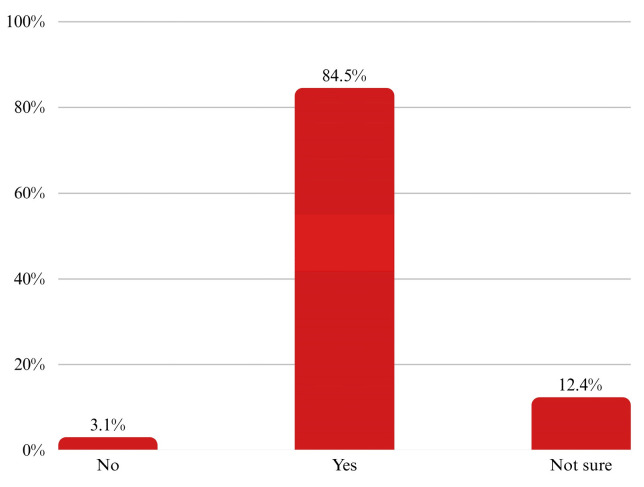
Regional distribution of colorectal cancer (CRC) knowledge and screening uptake among participants. Bars represent weighted proportions with 95% confidence intervals.

**Figure 2:**
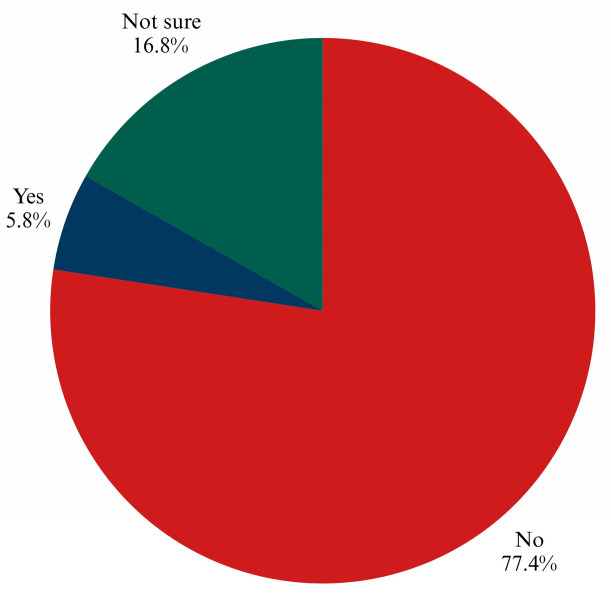
Predictors of fecal occult blood test (FOBT) uptake from logistic regression analysis, presented as odds ratios with 95% confidence intervals.

Only (21.7%) of respondents reported having received a physician’s recommendation for CRC screening, despite (84.5%) indicating willingness if such a recommendation were made (Figure 1), and 77.5% did not doubt the effectiveness of CRC screening (Figure 2).

Participants were asked to indicate their primary method of paying for healthcare (government sponsorship, private insurance, or personal payment) and, separately, to rate on a scale of 1–10 how burdensome they perceived healthcare costs to be, where 1 indicated ‘not burdensome at all’ and 10 indicated ‘extremely burdensome.

### 3.4. Barriers to the Examination of CRC and the Actual Practice of This Examination

**Figure 3:**
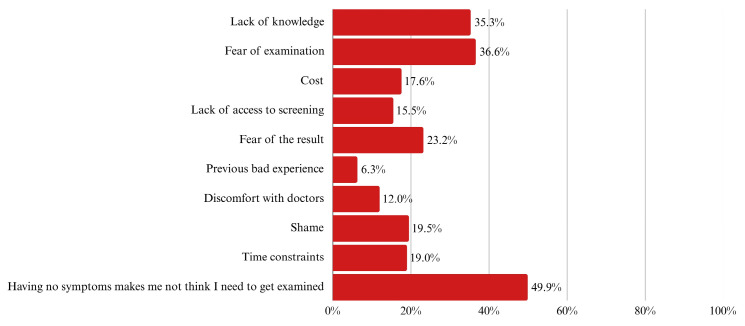
Reported barriers to CRC screening, stratified by screening modality. Categories include fear, lack of symptoms, cost, and limited physician recommendation.

The top-reported barrier was the perception that absence of symptoms eliminates the need for screening (49.9%), followed by fear of the procedure (36.6%) and lack of knowledge (35.3%) (Figure 3).

Among all participants, only 12.7% (73/573) had undergone a colonoscopy and 19.5% had completed a fecal occult blood test (FOBT). Among those who had never been screened (n = 461), the leading reason was “no symptoms” (42.9%). Among those who had undergone colonoscopy (n = 73), the most common timing of the last procedure was 2–5 years ago (34.2%) (Table 4).

Participants with a family history of CRC were more likely to cite fear of diagnosis (28.9% vs. 19.4%, *p* < 0.05), whereas those without family history more frequently reported lack of symptoms as a barrier.

### 3.5. Association of Knowledge About Early Screening of CRC with Demographic Information

Significant regional differences were observed in CRC knowledge levels (*p* < 0.001). Participants from the Southern region had the highest median knowledge score (5.0; IQR: 2.0–7.0) (Table 5).

### 3.6. Association of Having a Colonoscopy with Demographic Information

Participants aged ≥ 70 years had the highest rate of colonoscopy uptake (33.3%), making age a significant factor (*p* = 0.011). No statistically significant associations were found with gender, occupation, education, or income (Table 6).

In addition to FOBT predictors, logistic regression showed that higher education (OR = 1.64, 95% CI: 1.12–2.39) and family history of CRC (OR = 2.12, 95% CI: 1.35–3.31) were significant predictors of colonoscopy uptake.

### 3.7. Association of Having FOBT with Demographic Information

The highest rate of FOBT uptake was observed among individuals from the Southern region (42.6%, *p* < 0.001) and those aged 50–59 (28.6%, *p* < 0.001). The unemployed also had a significantly higher screening rate (38.9%, *p* = 0.020) (Table 7).

### 3.8. Predictors of FOBT Screening

Multivariate logistic regression revealed that participants aged 50–59 were nearly three times more likely to undergo FOBT compared to those aged 40–49 (OR = 2.79; 95% CI: 1.68–4.63; *p* < 0.001). Regionally, participants from the Southern region had the highest odds (OR = 8.97; 95% CI: 2.96–27.15; *p* < 0.001), followed by those from the Western region (OR = 3.15; 95% CI: 1.05–9.44; *p* = 0.040) compared to the Northern region (Table 8).

## 4. Discussion

### 4.1. Overview of Screening Uptake in Saudi Arabia

Colorectal cancer (CRC) continues to affect countless lives across the globe. Despite medical advances and clear guidelines, many people still do not undergo screening, often until it is too late. In Saudi Arabia, CRC stands as the most common cancer among men and the third among women. These numbers highlight the growing urgency to improve early detection through wider screening uptake. This study focused on adults aged 40 and above, aiming to understand how many undergo screening and what holds others back. Out of the 1210 individuals we initially approached, 573 were eligible and included; we excluded 637 due to being under the age limit or declining to participate, both consistent with guideline-based research practices. Our data showed that only 12.7% had ever undergone a colonoscopy, and just 19.5% had completed a Fecal Occult Blood Test (FOBT). Among those who had not been screened, the most common reasons were the absence of symptoms (49.9%), fear of the procedure (36.6%), and not knowing enough about screening (35.3%).

Compared to global averages, these rates are quite low. A large meta-analysis that reviewed hundreds of studies worldwide found much higher adherence to colonoscopy, 76.6% in observational studies and 80.4% in experimental settings [12]. Our numbers fall far below that benchmark. The reasons are not just about the availability of medical services. Nearly 90% of our participants said they had never even talked to a doctor about CRC screening. When that conversation does not happen, the idea of being screened may never occur to many people. The same global analysis showed that where people live, their income level, and even the year of the study can all influence screening rates [12]. The alarmingly low uptake of both colonoscopy (12.7%) and FOBT (19.5%) underscores a critical public health challenge. The most prominent barriers—absence of symptoms, fear of the procedure, and lack of knowledge—must be directly targeted in awareness campaigns and public health strategies. Addressing these barriers should be considered a national priority to reduce morbidity and mortality from colorectal cancer.

### 4.2. Regional Disparities

Looking within Saudi Arabia, we also found significant regional differences. For example, people from the Southern region were far more likely to have undergone FOBT compared to those from the Northern region (OR = 8.97) [9]. That echoes findings from Al-Baha, where willingness to be screened was common, but actual screening remained rare [9]. Similarly, in the Western region, only 38.7% had heard of screening, and most lacked even basic CRC knowledge [8]. In our sample, over 80% did not know how screening works, and more than half rated their knowledge level at the very bottom of the scale.

It is possible that the observed regional disparities in screening uptake partly reflect differences in population density and the concentration of tertiary healthcare facilities in larger urban centers, which may facilitate better access to screening services.

Our findings revealed that participants from the Southern region had significantly higher uptake of FOBT compared to other regions. Several factors may explain this pattern, including greater community health mobilization campaigns, targeted awareness initiatives, and stronger engagement of primary healthcare centers and physicians in promoting non-invasive tests. Localized cultural factors and community health worker involvement may also have fostered trust and increased acceptance of FOBT in this region. Conversely, lower uptake in peripheral regions may reflect disparities in healthcare infrastructure, availability of endoscopy services, and lower physician-to-population ratios. Future research should explore these enabling and limiting factors in more detail to inform scalable models for other regions.

These regional disparities indicate that interventions must be region-specific rather than uniform. For example, strategies in the Southern region, where FOBT uptake was highest, could be adapted and scaled to other regions, while in low-performing areas such as the Northern region, foundational awareness campaigns are urgently needed.

### 4.3. Knowledge and Awareness Gaps

This lack of awareness is a consistent barrier across different countries. In several African nations, studies found that limited patient education and a lack of guidance from healthcare providers severely reduced screening rates [13]. In Italy, women were much more likely to be screened if a doctor had talked to them about it [14]. Our results align with these findings. Only 10.6% of participants in our study had ever discussed CRC screening with their doctor, yet 84.5% said they would be willing to be screened if their physician recommended it. Our findings are consistent with regional evidence emphasizing the importance of physician engagement and culturally adapted interventions in promoting CRC screening. Alwassief et al. (2023) demonstrated that offering FIT testing in a population with a family history of colorectal neoplasia significantly increased colonoscopy uptake, suggesting that provider involvement is a key facilitator [15]. In addition, a Jordanian study by El Muhtaseb et al. (2025) identified fear, embarrassment, and a lack of information as significant barriers—especially among women—underscoring the need for culturally sensitive educational strategies [16]. Together, these findings strengthen our conclusion that physician advocacy coupled with culturally tailored outreach is essential for increasing CRC screening uptake in Saudi Arabia.

### 4.4. Financial, Emotional, and Cultural Barriers

Even with nearly half of the participants covered by government-sponsored healthcare, cost was still a concern. Around 17.6% said financial issues discouraged them from screening. This echoes findings from countries like Ghana and Nigeria, where the lack of insurance coverage or affordability has long been a barrier [17,18]. Sometimes, people do not know that government coverage includes these tests, or they may assume it does not.

Beyond the financials, emotional and psychological concerns also played a role. Many participants expressed fear about the procedure (23.2%), embarrassment (19.5%), or general discomfort around medical visits (12.0%). These reactions are not unique to Saudi Arabia. Studies from the Middle East and Africa have shown that cultural attitudes and fear of bad news can keep people away from early testing [13,18]. Beliefs like “I don’t have symptoms, so I don’t need a test” remain widespread. Nearly half of our participants agreed with this idea, a belief that also surfaced in other Saudi regions [8,9]. Changing that mindset requires campaigns that emphasize that CRC often develops silently and that screening is a proactive, not reactive, step.

Cultural norms and gender differences appear to influence screening behaviors. Women reported higher concerns about embarrassment and modesty, which are consistent with prior studies in Middle Eastern populations. Men, however, cited time constraints and perceptions of invulnerability as barriers. These findings highlight the importance of culturally sensitive awareness campaigns that address gender-specific concerns.

### 4.5. Education and Misconceptions

Another key insight from our data is that higher education does not always translate into better health knowledge. Although over 60% of our participants held university degrees, only 18.3% could name a CRC screening method. This pattern also appeared in studies from Egypt and Nigeria, where university graduates still lacked awareness of cancer prevention [13]. To close this gap, public health messages need to go beyond schools and universities and instead reach people where they are, through social media, public spaces, and healthcare settings.

We also found that people were more familiar with invasive methods like endoscopy than non-invasive ones like FIT. Stool tests were rarely mentioned. That contrasts with an extensive national study in Saudi Arabia, where people preferred FIT because it was less invasive and easier to complete [19]. This gap suggests we need to raise awareness about less invasive, accessible screening options, especially for people hesitant to undergo procedures like colonoscopy. Deibel et al. showed that when more people stick with a screening method, even if it is less sensitive, it can still reduce deaths more effectively than a highly sensitive test with low uptake [12].

Although both fecal occult blood test (FOBT) and fecal immunochemical test (FIT) are available, our survey data primarily captured FOBT use. FIT, which is now recommended due to higher sensitivity and ease of use, remains underutilized in Saudi Arabia, possibly due to limited awareness among the public and incomplete adoption of FIT in routine clinical practice.

### 4.6. Sociodemographic Influences

Age played a clear role in screening behavior. Participants aged 50–59 were nearly three times more likely to undergo FOBT compared to those aged 40–49 (OR = 2.79) [20]. This mirrors other studies, such as one among low-income Hispanic workers in the U.S., where older adults were more likely to follow CRC screening guidelines [21]. Interestingly, in our study, unemployed individuals had the highest screening rates for FOBT (38.9%). That is not what most international studies show, where employment and insurance typically correlate with better adherence. This could suggest that targeted outreach or time flexibility for this group may be having an impact.

One powerful theme that has emerged is the link between knowledge and motivation. People who strongly supported screening (scoring it 10 out of 10) were also the ones who rated themselves as more knowledgeable. Similar effects were seen in Africa, where education-focused interventions led to huge jumps in screening rates. One Egyptian study reported an increase from just 3.3% to over 60% after a brief campaign [13].

### 4.7. National Program and Policy Needs

All of these points point to the need for a national CRC screening program in Saudi Arabia. The low uptake rates and knowledge gaps we uncovered demand coordinated public health action. But the solutions shouldn’t be one-size-fits-all. People in different regions may need other types of outreach, such as mobile screening units in rural areas, digital reminders in urban centers, and public campaigns tailored to cultural beliefs. Global best practices show that combining easy access, doctor involvement, and simple communication can change habits and save lives [12,13,17].

By focusing on adults aged 40 and above, our study stayed in line with current screening guidelines. We excluded those younger or unwilling to participate, which helped sharpen the analysis. Still, as studies globally note rising rates of CRC in people aged 40–49, we may need to start looking even earlier in future work [12].

This research involved a broad national sample and followed solid methodological standards. By focusing on the right age group and including both univariable and multivariable analyses with 95% CIs, our findings strengthen the robustness and transparency of the reported associations. Few local studies have looked at both colonoscopy and FOBT in this detail or considered regional and personal factors so comprehensively. These strengths give healthcare leaders concrete data to build better strategies [20].

Saudi Arabia encounters a distinct problem in the context of CRC screening. Low awareness, limited doctor-patient communication, as well as emotional and cultural barriers, are still affecting the measure of this test. The disclosed results align with findings from other countries; yet, they differ in illustrating particular national and regional trends, necessitating tailored approaches to address these challenges. This study underscores the pressing necessity to allocate resources towards awareness initiatives, accessible and minimally intrusive testing alternatives, and healthcare systems that facilitate early detection. By tackling the appropriate issues with precise solutions, we might enable a greater number of individuals to gain from early diagnosis and eventually preserve lives.

One important insight in our study is the strong latent willingness to screen. Over 84% of participants expressed the intent to undergo CRC screening if their doctor recommended it, highlighting the pivotal role of healthcare providers. This emphasizes the potential impact of physician-led screening initiatives. However, physician–patient dialog on this topic remains minimal. Equipping and incentivizing physicians to routinely discuss CRC screening during primary care visits could therefore be one of the most effective strategies to raise national uptake rates.

From a policy perspective, several actionable recommendations emerge:

Establish a national CRC screening registry to systematically track adherence and outcomes.

Deploy mobile screening units in underserved or rural regions to improve access.

Integrate CRC reminders into electronic health records (EHRs) for adults aged 45+ to prompt primary care physicians during consultations.

Launch culturally appropriate media campaigns—especially targeting myths around symptoms, fear, and shame—to normalize screening.

Promote non-invasive testing options (e.g., FIT kits mailed to homes) to overcome hesitancy toward colonoscopy.

Policy recommendations are grounded in our findings: (1) increasing physician-led screening advocacy is supported by the 84.5% willingness rate if recommended by doctors; (2) reducing regional disparities aligns with observed variations in uptake between the Central and Southern regions; and (3) addressing reported barriers such as fear and lack of symptoms underpins the need for public awareness campaigns.

Interestingly, age influenced adherence: participants aged 50–59 were almost three times more likely to undergo FOBT compared to those aged 40–49 (OR = 2.79). This trend aligns with international findings and suggests that while guidelines now recommend screening starting at age 45, public awareness and provider outreach may still be lagging.

### 4.8. Strengths and Limitations

The study’s strength lies in its nationwide reach, robust methodology, and focus on both colonoscopy and FOBT. Compared to prior regional or small-scale investigations, our analysis offers a more comprehensive view of barriers across population subgroups in Saudi Arabia. Still, the data also highlight the urgent need for tailored, region-specific interventions. For example, the Southern region may benefit from scaling successful outreach models already in place, while the Northern region may require foundational awareness campaigns.

This study presents several limitations. Firstly, the cross-sectional nature of the research limits the ability to draw causal relationships between the identified factors and participation in colorectal cancer screening. Second, the use of an online self-administered survey with snowball sampling may have introduced selection bias toward more health-literate or digitally connected individuals, thereby limiting representativeness of the findings. In addition, since the data were self-reported, reporting bias may have influenced participants’ responses, particularly regarding their screening history and perceived barriers. Another limitation is that the sample was disproportionately weighted toward the Central region (62.1%), which may limit the generalizability of findings to other regions. This imbalance likely reflects higher survey reach through central academic and healthcare institutions. Thus, regional comparisons should be interpreted with caution, and future studies should employ stratified sampling to ensure proportional representation. Lastly, there may have been other influencing factors not measured in the study, such as prior exposure to health education or involvement in awareness campaigns.

## 5. Conclusions

In conclusion, bridging the gap between knowledge and action is key. Empowering both patients and physicians through structured programs and national policies can significantly improve CRC screening rates, potentially saving thousands of lives annually.

Future initiatives should not adopt a one-size-fits-all approach but should rather design region-specific interventions that address local cultural, socioeconomic, and healthcare system differences.

## Figures and Tables

**Table 1:** Demographic information.

Parameter	Category	N	%
Age	40–49	295	51.5%
	50–59	192	33.5%
	60–69	77	13.4%
	70 and more	9	1.6%
Gender	Female	273	47.6%
	Male	300	52.4%
Occupation	Student	8	1.4%
	Unemployed	18	3.2%
	Employee	380	66.8%
	Housewife	47	8.3%
	Retired	109	19.2%
	Other	7	1.2%
Educational level	Primary	11	1.9%
	Middle	23	4.0%
	Secondary	83	14.5%
	University	354	61.8%
	Post-graduate	102	17.8%
Monthly income	<5000 SAR	64	11.2%
	5000–10,000 SAR	97	16.9%
	10,000–20,000 SAR	288	50.3%
	>20,000 SAR	124	21.6%
Region	Northern	54	9.4%
	Southern	54	9.4%
	Middle	356	62.1%
	Eastern	14	2.4%
	Western	95	16.6%
How to pay for healthcare?	Private health insurance	153	26.7%
	Government sponsorship	270	47.1%
	Personal payment	147	25.7%
	Other	3	0.5%

**Table 2:** Medical history.

Parameter	Category	N	%
Do you have a first- or second-degree relative with colorectal cancer?	No	500	87.3%
	Yes	73	12.7%
Do you have any history of chronic gastrointestinal diseases?	No	479	83.6%
	Yes	94	16.4%
If you select Yes, state the disease	Esophageal problems	7	8.0%
	Gastric problems	16	18.4%
	Intestinal problems	56	64.4%
	Other	16	18.4%
Have you discussed colorectal cancer with your doctor?	No	512	89.4%
	Yes	61	10.6%

**Table 3:** Knowledge and attitude towards colorectal cancer (CRC).

Parameter	Category	N	%
How would you rate your knowledge of early screening for colorectal cancer?	1	298	52.0%
	2	34	5.9%
	3	42	7.3%
	4	41	7.2%
	5	64	11.2%
	6	23	4.0%
	7	24	4.2%
	8	16	2.8%
	9	10	1.7%
	10	21	3.7%
What is the benefit of early screening for colorectal cancer?	Early detection of cancer	329	57.4%
	Preventing the development of cancer	262	45.7%
	I do not know	102	17.8%
	Other	5	0.9%
In your opinion, what is the appropriate age to start colorectal cancer screening for healthy people?	<20	43	7.5%
	20–30	87	15.2%
	31–40	188	32.9%
	41–50	144	25.2%
	>50	51	8.9%
	Other	23	4.0%
	I do not know	36	6.3%
Do you know how to screen for colorectal cancer?	No	468	81.7%
	Yes	105	18.3%
If yes, what methods do you know?	Endoscopy	41	41.0%
	Stool analysis	12	12.0%
	Sample	2	2.0%
	Radiographic examination	1	1.0%
	2 or more of these methods	38	38.0%
	Other	6	6.0%
Do you think you should be screened for colorectal cancer even if you do not have symptoms?	No	194	33.9%
	Yes	379	66.1%
How to pay for healthcare?	1	41	7.2%
	2	9	1.6%
	3	19	3.3%
	4	21	3.7%
	5	62	10.8%
	6	27	4.7%
	7	32	5.6%
	8	45	7.9%
	9	25	4.4%
	10	292	51.0%

Note: For the question on healthcare costs, values 1–10 represent perceived financial burden, from 1 = not burdensome to 10 = extremely burdensome.

**Table 4:** Barriers against the examination of CRC and the actual practice of this examination.

Parameter	Category	N	%
Barriers to colorectal cancer examination	Lack of knowledge	202	35.3%
	Fear of examination	210	36.6%
	Cost	101	17.6%
	Lack of access to screening	89	15.5%
	Fear of the result	133	23.2%
	Previous bad experience	63	6.3%
	Discomfort with doctors	69	12.0%
	Shame	112	19.5%
	Time constraints	109	19.0%
	Having no symptoms makes me think I do not need to be examined	286	49.9%
Have you had a colonoscopy before?	No	500	87.3%
	Yes	73	12.7%
If yes, when was your last checkup?	1–3 months	9	12.3%
	4–6 months	5	6.8%
	7 months–1 year	12	16.4%
	2–5 years	25	34.2%
	More than 5 years	22	30.1%
Have you ever had a fecal occult blood test?	No	461	80.5%
	Yes	112	19.5%
If you have not had any examinations, what is the reason?	No symptoms	81	42.9%
	Negligence	10	5.3%
	No need for that	32	16.9%
	No request from the doctor	14	7.4%
	I do not know	16	8.5%
	Other	36	19.0%

**Table 5:** Association of knowledge about early screening of CRC with demographic information.

Parameter	Category	Median (IQR)	*p* Value
Age	40–49	1.0 (1.0–4.0)	0.067
	50–59	2.5 (1.0–5.0)	
	60–69	1.0 (1.0–5.0)	
	70 and more	1.0 (1.0–4.5)	
Gender	Female	1.0 (1.0–5.0)	0.690
	Male	1.0 (1.0–5.0)	
Occupation	Student	2.5 (1.0–5.75)	0.150
	Unemployed	2.5 (1.0–5.25)	
	Employee	1.0 (1.0–5.0)	
	Housewife	1.0 (1.0–3.0)	
	Retired	1.0 (1.0–5.0)	
	Other	4.0 (3.0–5.0)	
Educational level	Primary	1.0 (1.0–7.0)	0.675
	Middle	1.0 (1.0–6.0)	
	Secondary	1.0 (1.0–4.0)	
	University	1.0 (1.0–5.0)	
	Post-graduate	2.0 (1.0–5.0)	
Monthly income	<5000 SAR	1.0 (1.0–4.0)	0.539
	5000–10,000 SAR	1.0 (1.0–5.0)	
	10,000–20,000 SAR	1.0 (1.0–5.0)	
	>20,000 SAR	1.0 (1.0–5.0)	
Region	Northern	1.0 (1.0–5.0)	<0.001
	Southern	5.0 (2.0–7.0)	
	Middle	1.0 (1.0–4.0)	
	Eastern	2.5 (1.0–5.25)	
	Western	1.0 (1.0–4.0)	
How to pay for healthcare?	Private health insurance	1.5 (1.0–5.0)	0.478
	Government sponsorship	1.0 (1.0–5.0)	
	Personal payment	1.0 (1.0–4.0)	
	Other	4.0 (4.0-not mentioned)	

**Table 6:** Association of having a colonoscopy with demographic information.

Parameter	Category	No		Yes		*p* Value
		N	%	N	%	
Age	40–49	268	90.8%	27	9.2%	0.011
	50–59	158	82.3%	34	17.7%	
	60–69	68	88.3%	9	11.7%	
	70 and more	6	66.7%	3	33.3%	
Gender	Female	235	86.1%	38	13.9%	0.419
	Male	265	88.3%	35	11.7%	
Occupation	Student	8	100.0%	0	0.0%	0.743
	Unemployed	15	83.3%	3	16.7%	
	Employee	332	87.4%	48	12.6%	
	Housewife	40	85.1%	7	14.9%	
	Retired	94	86.2%	15	13.8%	
	Other	7	100.0%	0	0.0%	
Educational level	Primary	8	72.7%	3	27.3%	0.136
	Middle	18	78.3%	5	21.7%	
	Secondary	76	91.6%	7	8.4%	
	University	313	88.4%	41	11.6%	
	Post-graduate	85	83.3%	17	16.7%	
Monthly income	<5000 SAR	57	89.1%	7	10.9%	0.867
	5000–10,000 SAR	83	85.6%	14	14.4%	
	10,000–20,000 SAR	250	86.8%	38	13.2%	
	>20,000 SAR	110	88.7%	14	11.3%	
Region	Northern	46	85.2%	8	14.8%	0.793
	Southern	47	87.0%	7	13.0%	
	Middle	314	88.2%	42	11.8%	
	Eastern	13	92.9%	1	7.1%	
	Western	80	84.2%	15	15.8%	
How to pay for healthcare?	Private health insurance	129	84.3%	24	15.7%	0.201
	Government sponsorship	233	86.3%	37	13.7%	
	Personal payment	135	91.8%	12	8.2%	
	Other	3	100.0%	0	0.0%	

**Table 7:** Association of having FOBT with demographic information.

Parameter	Category	No		Yes		*p* Value
		N	%	N	%	
Age	40–49	257	87.1%	38	12.9%	<0.001
	50–59	137	71.4%	55	28.6%	
	60–69	60	77.9%	17	22.1%	
	70 and more	7	77.8%	2	22.2%	
Gender	Female	223	81.7%	50	18.3%	0.478
	Male	238	79.3%	62	20.7%	
Occupation	Student	7	87.5%	1	12.5%	0.020
	Unemployed	11	61.1%	7	38.9%	
	Employee	309	81.3%	71	18.7%	
	Housewife	44	93.6%	3	6.4%	
	Retired	80	73.4%	29	26.6%	
	Other	6	85.7%	1	14.3%	
Educational level	Primary	7	63.6%	4	36.4%	0.277
	Middle	19	82.6%	4	17.4%	
	Secondary	71	85.5%	12	14.5%	
	University	287	81.1%	67	18.9%	
	Post-graduate	77	75.5%	25	24.5%	
Monthly income	<5000 SAR	55	85.9%	9	14.1%	0.351
	5000–10,000 SAR	82	84.5%	15	15.5%	
	10,000–20,000 SAR	225	78.1%	63	21.9%	
	>20,000 SAR	99	79.8%	25	20.2%	
Region	Northern	49	90.7%	5	9.3%	<0.001
	Southern	31	57.4%	23	42.6%	
	Middle	294	82.6%	62	17.4%	
	Eastern	12	85.7%	2	14.3%	
	Western	75	78.9%	20	21.1%	
How to pay for healthcare?	Private health insurance	117	76.5%	36	23.5%	0.051
	Government sponsorship	218	80.7%	52	19.3%	
	Personal payment	125	85.0%	22	15.0%	
	Other	1	33.3%	2	66.7%	

**Table 8:** Predictors of FOBT screening.

Parameter	Category	OR	95% CI		*p* Value
			LB	UB	
Age	40–49	Ref.	Ref.	Ref.	Ref.
	50–59	2.791	1.684	4.626	<0.001
	60–69	1.410	0.631	3.150	0.402
	70 and more	1.709	0.301	9.710	0.546
Occupation	Student	Ref.	Ref.	Ref.	Ref.
	Unemployed	6.554	0.570	75.346	0.131
	Employee	2.694	0.285	25.453	0.387
	Housewife	0.609	0.048	7.753	0.702
	Retired	3.447	0.339	35.033	0.296
	Other	1.861	0.074	47.037	0.706
Region	Northern	Ref.	Ref.	Ref.	Ref.
	Southern	8.966	2.962	27.145	<0.001
	Middle	2.072	0.774	5.542	0.147
	Eastern	1.800	0.297	10.901	0.522
	Western	3.150	1.052	9.439	0.040

Note: ‘Ref.’ indicates the reference category used in logistic regression, here representing the age group 40–49, student occupation, and the Northern region.

## Data Availability

The data presented in this study are available upon reasonable request from the corresponding author. The data are not publicly available due to ethical and privacy restrictions, in accordance with the regulations approved by the Institutional Review Board of Imam Mohammad Ibn Saud Islamic University (IRB Reference: HAPO-01-R-061).

## Abbreviations

The following abbreviations are used in this manuscript:

CRC	Colorectal cancer
FOBT	Fecal occult blood test
IARC	International Agency for Research on Cancer
mCRC	Metastatic CRC
ASIR	Age-standardized incidence rate
ASMR	Age-standardized mortality rate
SSLs	Sessile Serrated Lesions
